# The Paracrine Effect of Adipose-Derived Stem Cells Orchestrates Competition between Different Damaged Dermal Fibroblasts to Repair UVB-Induced Skin Aging

**DOI:** 10.1155/2020/8878370

**Published:** 2020-12-17

**Authors:** Feng Qin, Jiuzuo Huang, Wenchao Zhang, Mingzi Zhang, Zhenjiang Li, Loubin Si, Xiao Long, Xiaojun Wang

**Affiliations:** ^1^Department of Plastic Surgery, Peking Union Medical College Hospital, Peking Union Medical College, Beijing 100032, China; ^2^Department of Vascular Surgery, The First Affiliated Hospital of the Medical School of Zhejiang University, Hangzhou 310000, China

## Abstract

**Background:**

Human dermal fibroblasts (HDFs) are the primary cells in skin and are associated with UVB-induced skin photoaging. Adipose-derived stem cells (ASCs) have been proposed as a treatment for skin aging. The goal of this study was to investigate paracrine mechanisms by which ASCs repair HDFs damage from UVB exposure.

**Methods:**

ASCs were cocultured with UVB-irradiated and nonirradiated HDFs. We compared HDF senescence, proliferation, migration, oxidative stress, and cytokine expression. In a nude mouse UVB-induced photoaging model, ASCs were injected subcutaneously, and skin samples were collected weekly between postoperative weeks 3 through 7. Histological analysis, PCR, ELISA, and immunohistochemistry were used to analyze the effect of ASCs.

**Results:**

Compared with UVB-irradiated HDFs, nonirradiated HDFs showed higher proliferation and migration, reduced apoptosis, and fewer senescent cells when cocultured with ASCs. The expression of extracellular matrix-related cytokines was also regulated by ASCs. In addition, ASCs effectively reversed UVB-induced skin photoaging in vivo. We propose that ASCs more robustly coordinate healthy HDFs than UVB-damaged HDFs to repair aging skin.

**Conclusions:**

ASCs improved the function of both UVB-damaged and healthy HDFs through paracrine effects. However, the impact of ASCs on healthy HDFs was greater than UVB-damaged HDFs. These findings help to elucidate the underlying mechanisms of the skin rejuvenation effect of ASCs.

## 1. Introduction

With the development of the economy and aging of the population, skin aging is receiving increased attention from plastic surgeons. Human dermal fibroblasts (HDFs) are the primary cell type in the dermis and are responsible for extracellular matrix (ECM) deposition and remodeling [1], supplying skin with structural integrity and elasticity. In the process of skin aging, the quantity and proliferation rates of HDFs are declined, and collagen is reduced. On the other hand, matrix-degrading metalloproteinases (MMPs) are increased, degrading and changing the structure of the ECM, which accelerates the breakdown of connective tissue. All of these changes result in the thinning of the dermis, enhancement of wrinkles, and loss of elasticity. Skin aging is caused by intrinsic factors (e.g., time, genetic factors, and hormones) and extrinsic factors (e.g., ultraviolet (UV) exposure and pollution). Eighty percent of skin aging primarily results from exposure to UV light, which is known as photoaging [2]. Ultraviolet B (UVB) radiation penetrates the epithelial layer and causes deoxyribonucleic acid (DNA) damage in the dermis of the skin [3]. However, most UVB radiation directly affects cells in the upper dermis [4]. Compared with HDFs in the lower dermis, more HDFs in the upper dermis suffer UVB-induced DNA damage. Failure of aged-cellular repair results in cell death. Health cells are gradually generated to replace the damaged cells to keep homeostasis.

Currently, some clinical studies have shown that autologous fat grafting, nanofat, and adipose stromal cells reduce wrinkles, increase dermal thickness, improve skin elasticity, and whiten skin [5–8]. Adipose-derived stem cells (ASCs) play an important role in these therapies. ASCs have the ability to differentiate into different cell lineages, such as adipocytes, endothelial cells (ECs), osteocytes, cardiomyocytes, and neurons [9]. In addition, ASCs secrete various biologically active molecules to repair damaged neighboring cells and influence the surrounding microenvironment [10]. ASCs are considered a promising tool for cell-based therapy, especially in skin rejuvenation, wound healing, and scar remodeling [11]. Because ASCs are usually injected into the subcutaneous fat layer, the paracrine effect of ASCs is the main mechanism by which skin rejuvenation occurs [12]. However, the role of the paracrine effect of ASCs on the repair of different UVB-induced damaged HDFs is still unknown.

We hypothesized that ASCs could repair different UVB-damaged HDFs to various degrees via paracrine factors. To induce photoaging and natural aging in vitro and in vivo, HDFs and nude mice were irradiated with UVB light, and the control group received no irradiation. We found that ASCs enhanced HDF cell function. However, nonirradiated HDFs were more robust than UVB-irradiated HDFs after coculture with ASCs. This finding suggests that ASC treatment may more strongly impact HDFs in the lower dermis.

## 2. Methods

### 2.1. Isolation, Culture, and Characterization of ASCs

Adipose tissue was collected from liposuction surgeries and used to isolate ASCs. Adipose tissue was cut into small pieces, digested with 1 mg/ml collagenase type I with agitation at 200 rpm at 37°C for 45 min. After that, the samples were centrifuged at 1200 rpm for 10 min at room temperature. The cell pellets were collected and resuspended in 10 ml of erythrocyte lysis buffer to lyse the red blood cells (RBCs). After an additional centrifugation, DMEM/F12 (HyClone, USA) containing 10% fetal bovine serum (FBS) (Yeasen, China) was added, and 100 *μ*m filters (BD Falcon, San Jose, CA, USA) were used to obtain the cell suspension. The cell pellet was resuspended in ASC plating medium and incubated at 37°C in a humidified 5% CO_2_ atmosphere. After a 24-hr incubation period, nonadherent cells were removed, and the cell medium was replaced with fresh ASC plating medium. Experiments were performed when the cells were at passages 2-4.

ASCs were characterized by differentiation potential and cell surface markers [13]. ASCs were stained with fluorescein isothiocyanate- (FITC-) and phycoerythrin- (PE-) conjugated monoclonal antibodies against human CD45, CD90, CD44, and CD29. The cells were subsequently analyzed by flow cytometry (BeamCyte, Changzhou, China). ASCs were incubated in adipogenic or osteogenic medium to evaluate their multidifferentiation potential. The cells were seeded in a 6-well plate at a density of 1 × 10^5^ cells per well. After culturing the ASCs in differentiation media for 21 days, adipocyte differentiation was assessed using oil red O staining as an indicator of intracellular lipid accumulation, and osteogenic differentiation was assessed using Alizarin red staining.

### 2.2. Transwell Coculture of ASCs and HDFs

**Figure 1:**
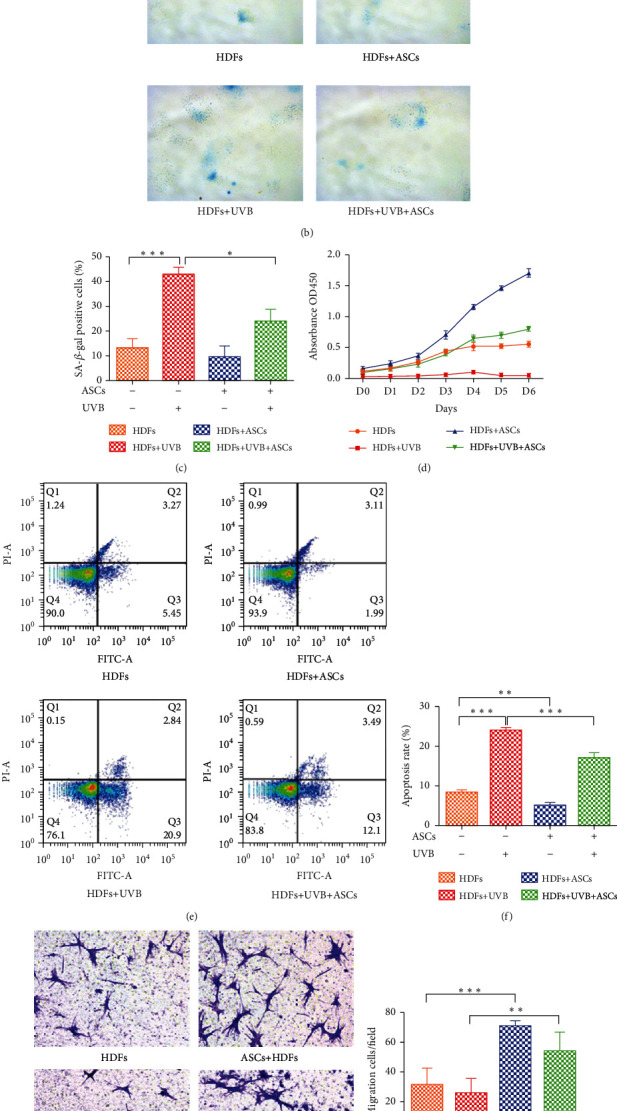
Assessment of the functions of HDFs cocultured with ASCs. (a) Schematic illustration of the cell experiment. (b) Representative images of senescence-associated *β*-galactosidase (SA-*β*-gal) staining. Positive blue staining of SA-*β*-gal appeared in senescent HDFs. (c) The average percentage of SA-*β*-gal-positive cells was quantified. Five random fields were selected for counting. (d) Proliferation of HDFs was assessed by CCK-8 assays. (e) HDF apoptosis was measured by FACS analysis after Annexin V-FITC/PI staining. (f) The apoptosis rates of HDFs were quantified. (g) Representative images of migrated HDFs (magnification, ×200). (h) The number of migrated HDFs per field. Five random fields were selected for counting. All experiments were independently conducted in triplicate, and the values are expressed as the mean ± SD. (^∗^*p* < 0.05, ^∗∗^*p* < 0.01, ^∗∗∗^*p* < 0.001).

To evaluate the paracrine effect of ASCs on HDFs, ASCs and HDFs were cocultured in a Transwell system. HDFs were purchased from BNCC Beijing, China (Catalog No: 340397) and were cultured with or without UVB treatment. UVB treatment of HDFs was performed as previously described [14]. After 4 days of culture, HDFs were trypsinized and seeded on the surface of 24-well plates at a density of 1 × 10^4^ cells/well. ASCs were dispensed into the upper chamber containing 0.4 *μ*m pores (Corning Costar, USA), at a density of 5 × 10^3^ cells/well. Four coculture groups are shown in Figure 1(a). Cells were cocultured for 3 days in DMEM/F12 with 10% FBS. HDFs grown in the lower chambers were collected for subsequent experiments.

### 2.3. Senescence-Associated *β*-Galactosidase (SA-*β*-gal) Staining

SA-*β*-gal staining is widely used as a biomarker of cellular senescence, and the cells were stained using a senescence-associated *β*-gal staining kit (Beyotime, Shanghai, China) according to the manufacturer's protocol. Senescent cells were identified by blue staining. The number of blue cells (SA-*β*-gal-positive cells) in five randomly selected fields of view in each sample was counted under a microscope, and the experiment was performed three independent times.

### 2.4. Apoptosis Analysis

Apoptosis was determined using an Annexin V-FITC/propidium iodide (PI) detection kit (Beijing 4A Biotech Co., China) and flow cytometry. HDFs were harvested and washed twice with ice-cold PBS and resuspended in binding buffer (1 × 10^6^ cells/ml). Annexin V-FITC and PI were added and incubated. The samples were scanned and analyzed by flow cytometry (BeamDiag, Changzhou, China).

### 2.5. Proliferation Assay

HDF proliferation was assessed using the cell counting kit-8 assay (CCK-8; Dojindo, Tokyo, Japan) according to the manufacturer's instructions. HDFs (5 × 10^3^ cells/well) were seeded onto 96-well plates. Following one day of culture, cell proliferation was measured for 6 days. Absorbance was measured at a wavelength of 450 nm using a microplate spectrophotometer (Bio-Rad, California, USA).

### 2.6. Migration Assay

The migration capacity of HDFs was evaluated using 24-well Transwell membrane filters with 8 *μ*m pores (Corning Costar, USA). HDFs (1 × 10^5^ cells/well) in serum-free medium were added to the upper chamber. Medium with 10% FBS was placed in the bottom chamber. After incubation at 37°C for 24 h, the upper side of the filter was cleaned by a cotton-tipped swab. Cells were then fixed, stained with Giemsa, and imaged with a light microscope (Leica Microsystems, Germany). Five randomly selected fields were photographed and counted for each sample.

### 2.7. Reactive Oxygen Species (ROS) Assay

ROS generation was measured by the 2,7-dichlorodihydrofluorescein diacetate (DCFH-DA) probe (Jiancheng, Nanjing, China). After HDFS received UVB treatment, DCFH-DA (10 *μ*M) was added to the medium for 45 min at 37°C in the dark. The fluorescence intensity of DCFH-DA was measured and calculated using flow cytometry (BeamDiag, Changzhou, China), and intracellular ROS levels were monitored in the FITC channel.

### 2.8. Western Blotting

Protein samples were extracted from HDFs or skin tissues, subjected to sodium dodecyl sulfate-polyacrylamide gel electrophoresis (SDS-PAGE), and transferred to a polyvinylidene difluoride membrane. Membranes were blocked with TBST containing 5% nonfat milk for 1 h and then incubated with primary antibodies overnight at 4°C. The membranes were then incubated with secondary antibodies, and the protein bands were detected by enhanced chemiluminescence (Bio-Rad, USA). All western blot values were initially standardized to the corresponding *β*-actin band prior to comparative analyses.

### 2.9. Quantitative Reverse Transcription-Polymerase Chain Reaction (qRT-PCR)

Total RNA was extracted using TRIzol reagent (Invitrogen, Shanghai, China) according to the manufacturer's instructions. RNA was reverse transcribed into cDNA using SuperScript III First-Strand Synthesis SuperMix (Invitrogen, USA). qRT-PCR was performed on QuantStudio Real-Time PCR Systems (Applied Biosystems, USA) using SYBR Select Master Mix (Invitrogen, USA). The primers (Supplementary Table 1) were synthesized by Sangon Biotech (Shanghai, China). The relative mRNA expression was calculated by the 2-*ΔΔ*Ct method, with *β*-actin housekeeping gene.

### 2.10. Enzyme-Linked Immunosorbent Assay (ELISA)

The levels of growth factors (MMP-1, TGF-*β*, EGF, and IGF-1) and inflammatory factors (IL-6 and IL-10) in the supernatants of HDFs and skin tissue from each group were detected by ELISA kits (Cloud-Clone Corp, China) according to the manufacturer's instructions. The OD450 was calculated by subtracting the background value, and standard curves were plotted.

### 2.11. Animal Experiments

**Figure 2:**
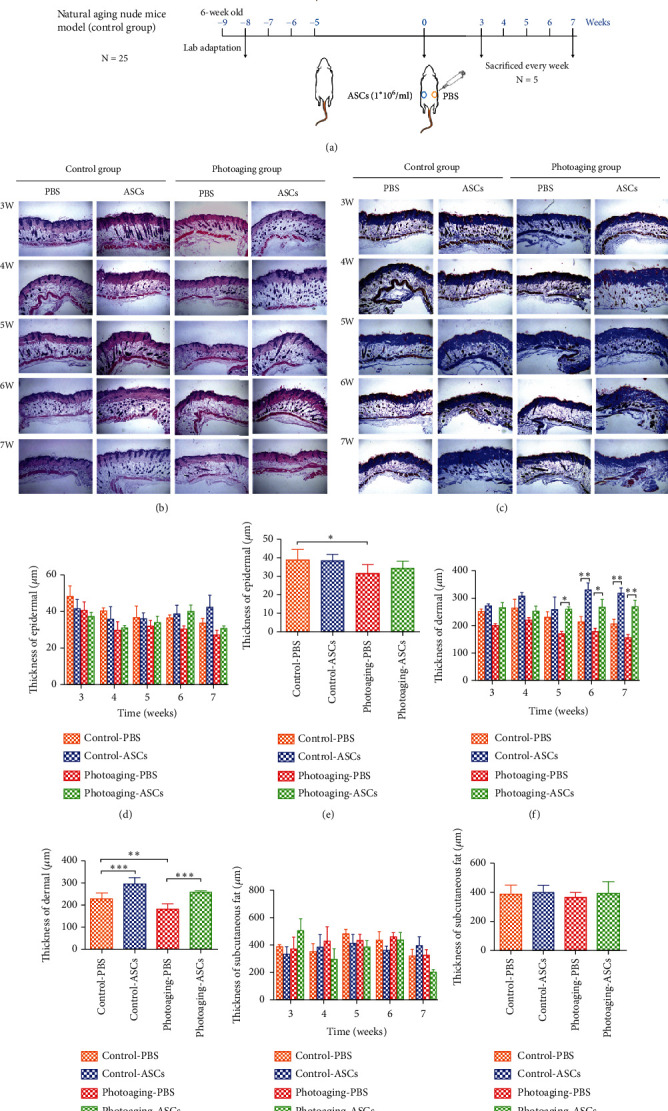
Histological analysis of the mice skin. (a) Schematic illustration of the in vivo study. (b) H&E staining. (c) Masson's trichrome staining. (d, e) Epidermal thickness analysis. (f, g) Dermal thickness analysis. (h, i) Subcutaneous fat thickness analysis. *n* = 25 (25 mice per group, 3 areas analyzed for each sample).

Nude mice were acclimatized to the laboratory conditions for 1 week prior to the beginning of the experiments. The mice were randomly divided into 2 groups (UVB-induced photoaging nude mouse model group and control group, *n* = 25/group). UVB-induced photoaging was performed as previously described [15]. A UVB lamp was used to irradiate mice five times a week for 8 weeks. The irradiation dose was 160 mJ/cm^2^ in the first week, followed by 210, 280, and 370 mJ/cm^2^ in the second to fourth weeks, respectively, and 370 mJ/cm^2^ in the fifth to eighth weeks. The total UVB dose was approximately 80 MED (12.7 J/cm^2^). The control group did not receive any treatment. The animal experimental design is shown in Figure 2(a). After the mouse models were established after eight weeks, 200 *μ*l of either PBS (control) or ASCs in PBS (1 × 10^6^) was administered bilaterally on the mouse dorsum. For each group, 5 mice were sacrificed at weeks 3, 4, 5, 6, and 7 postinjection.

### 2.12. Histological Analysis

For histological observation, skin tissue samples were harvested, fixed in 4% paraformaldehyde for 1 day, and embedded in paraffin blocks. Microtome sections (4 *μ*m) of tissue adherent on glass slides were dewaxed and rehydrated according to standard procedures. Slides were stained with hematoxylin and eosin (H&E) and Masson's trichrome. Photomicrographs were taken using a light microscope (Leica Microsystems. German). The skin thickness of each sample was measured using the ImageJ software (NIH, Bethesda, MD) in three randomly selected fields.

### 2.13. Immunohistochemical (IHC) Staining

Tissue sections were immersed in 3% H_2_O_2_, and a microwave antigen retrieval step was performed. The sections were blocked with BSA and then incubated with primary antibodies against MMP-1, TGF-*β*, EGF, IGF-1, and Nrf2 (Abcam, Cambridge, MA, USA) overnight at 4°C. The slides were washed with PBS and incubated with secondary antibody for 20 min. Then, DAB staining was performed, and slides were counterstained with hematoxylin. Images were obtained under 100x and 200x magnification.

### 2.14. Statistical Analysis

All values are presented as the means ± standard deviation (SD). The significance of differences among each group was analyzed by one-way ANOVA or two-way ANOVA using GraphPad Prism 7.0 (GraphPad Software, USA). Differences were considered significant at *p* values < 0.05.

## 3. Results

### 3.1. Phenotypic Characterization and Multipotency of ASCs

**Figure 3:**
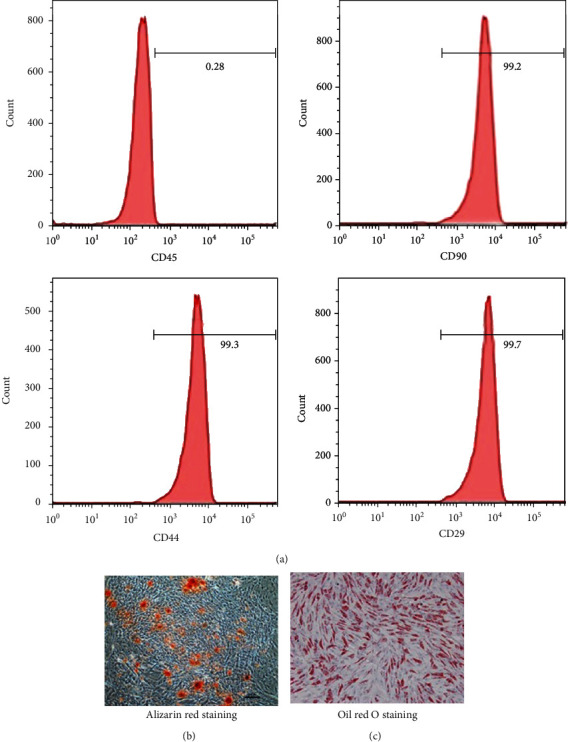
Characterization of ASCs by surface marker phenotype and differentiation potential. ASCs were assessed for the presence of mesenchymal stem cell properties. (a) Immunophenotype assessment of ASCs via flow cytometry analysis of the percentage of positive mesenchymal stem cell markers (CD90, CD44, and CD29) and negative hematopoietic markers (CD45). (b) Osteogenic-differentiated cells were analyzed by Alizarin red staining. (c) Adipogenic-differentiated cells were analyzed by oil red O staining.

Culture expanded ASCs exhibited expression profiles characteristic of mesenchymal stem cells (MSCs) (positive for CD90, CD44, and CD29; negative for CD45) (Figure 3(a)). To evaluate the multipotency capacity of ASCs, the ASCs were induced differentiation into osteocytes and adipocytes. Osteocytes and adipocytes were stained with Alizarin red and oil red O, respectively (Figures 3(b) and 3(c)). These results indicate that isolated ASCs maintained MSC characteristics.

### 3.2. Effects of ASCs on HDF Senescence, Proliferation, Apoptosis, and Migration

SA-*β*-gal was used to directly stain senescent cells. We observed an increase in the percentage of SA *β*-gal-positive cells (bluish color) in the UVB irradiation group compared to the nonirradiated group (*p* < 0.0001). ASCs decreased the number of senescent cells in the UVB-irradiated HDF group (*p* < 0.05). However, there was no significant difference in the nonirradiated HDF group when cocultured with ASCs (Figures 1(b) and 1(c)). These results suggest that ASCs effectively suppressed UVB-induced senescence.

HDFs play a key role in controlling skin physiology, and their proliferation and apoptosis are also critical for structural homeostasis [16]. The CCK-8 assay confirmed that UVB irradiation significantly decreased HDF proliferation compared to nonirradiated cells. ASC coculture improved the proliferation of UVB-irradiated HDFs. Nonirradiated HDFs cocultured with ASCs had the highest cell proliferation capacity (Figure 1(d)).

As shown in Figures 1(e) and 1(f), UVB radiation significantly increased HDF apoptosis rate from 8.28% to 23.93% (*p* < 0.001). ASCs decreased the apoptosis rate in both nonirradiated HDFs (5.08%) and UVB-irradiated HDFs (16.99%).

It is well accepted that HDF migration is vital in skin tissue regeneration and wound healing [17]. The migration of HDFs was investigated using a Transwell assay. No significant differences were observed in the UVB-irradiated HDF group compared to control, whereas coculture with ASCs equally promoted HDF migration in both the UVB-irradiated group and the nonirradiated group (53.8% and 71.0%, respectively) (Figures 1(g) and 1(h)).

### 3.3. Effects of ASCs on HDF Oxidative Stress

**Figure 4:**
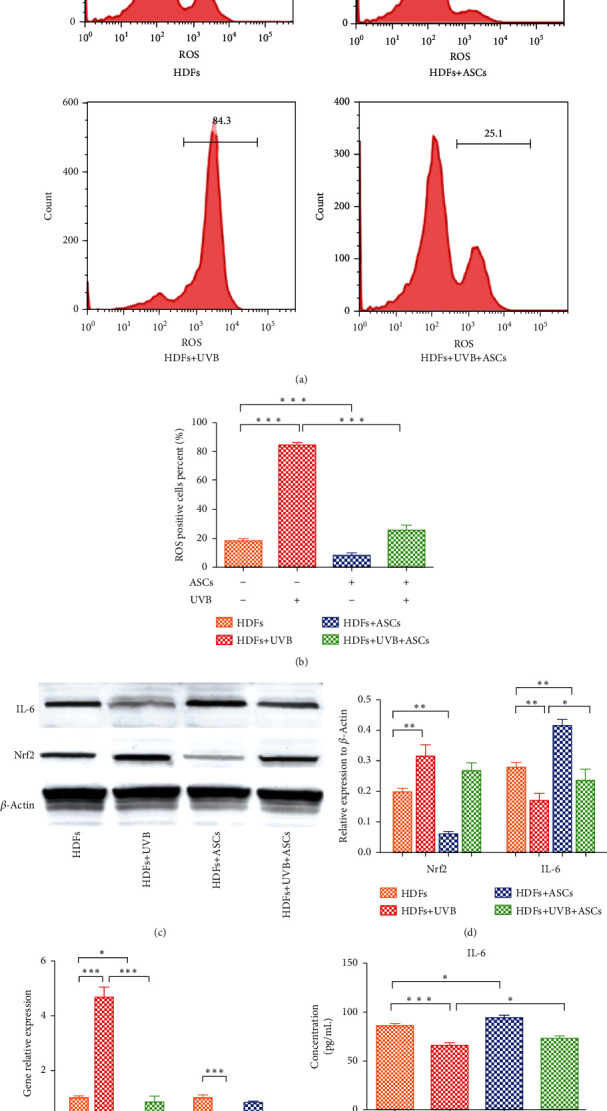
Assessment of oxidative stress in HDFs cocultured with ASCs. (a) Flow cytometry showing the reactive oxygen species (ROS) levels in HDFs. (b) Quantification of ROS-positive cells. (c) Western blots showing Nrf2 and IL-6 expression in HDFs. (d) Western blots quantification of Nrf2 and IL-6. (e) RT-PCR analysis of Nrf2 and IL-6 gene expression in HDFs. (f) ELISA results showing the expression of IL-6 in the cell culture supernatant.

To confirm whether ASCs exerted a protective effect on HDFs by antioxidative actions, we measured the levels of reactive oxygen species (ROS). Compared to nonirradiated HDFs, UVB-irradiated HDFs had increased ROS levels (*p* < 0.0001). Coculture with ASCs significantly decreased the number of ROS-positive cells in the nonirradiated HDF group (17.7% vs. 8.0%) and the UVB-irradiated HDF group (83.93% vs. 25.7%) (Figures 4(a) and 4(b)). Nuclear factor erythroid 2-related factor (Nrf2) protein expression and Nrf2 target gene expression were increased in the UVB-irradiated HDF group (Figures 4(c)–4(e)). HDFs cocultured with ASCs had reduced the expression of Nrf2, indicating reduced oxidative stress-induced injury. We further investigated the expression of IL-6 in HDFs. In the UVB-irradiated group, the gene and protein levels of IL-6 were significantly reduced in HDFs (Figures 4(c)–4(e)). The protein expression of IL-6 was significantly increased when HDFs were cocultured with ASCs, while the gene expression of IL-6 showed no difference in either the UVB-irradiated group or the nonirradiated group when the HDFs were cocultured with ASCs. We also observed similar changes in the cell culture supernatants (Figure 4(f)).

### 3.4. Effects of ASCs on HDF Expression of Cytokines Involved in ECM Remodeling and Skin Aging

**Figure 5:**
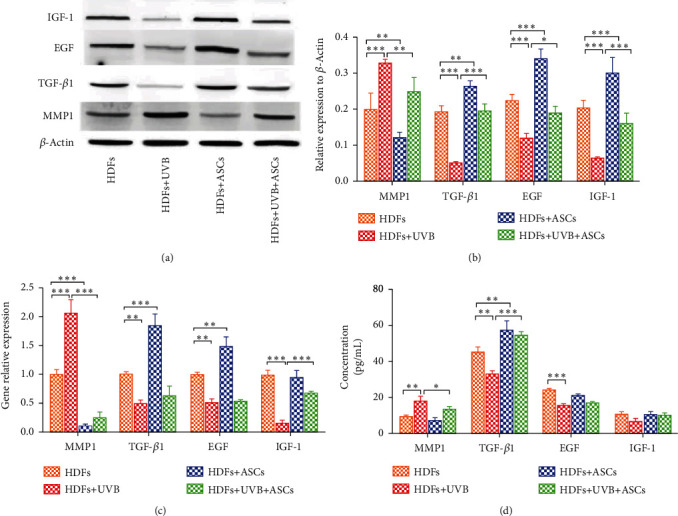
Cytokine expression involved in ECM remodeling and skin aging. (a) Western blots showing cytokine expression in HDFs. (b) Western blots quantitative analysis of cytokine expression. (c) RT-PCR showing the gene expression of cytokines in HDFs. (d) The expression of cytokines in the cell culture supernatant.

The effect of ASCs on HDF expression of cytokines was examined by western blotting and qPCR. We also measured the levels of these cytokines in the cell culture medium using ELISA. UVB irradiation leads to increased expression of matrix metalloproteinase 1 (MMP1). Coculture with ASCs decreased HDF expression of MMP1, which reduced the degradation of collagen (Figures 5(a)–5(c)). The expression of tumor growth factor (TGF)-*β*1 was significantly reduced in the UVB-irradiated HDF group, and coculture with ASCs promoted expression of TGF-*β*1 and increased collagen synthesis (Figures 5(a) and 5(b)). However, PCR analysis showed that coculture with ASCs only significantly increased the gene expression of TGF-*β*1 in the nonirradiated group (*p* < 0.05), and there was no significant change in the UVB-irradiated group (Figure 5(c)). ELISA results showed that the expression of TGF-*β*1 was significantly increased in the cell supernatant when HDFs were cocultured with ASCs in both the UVB-irradiated HDF group and the nonirradiated HDF group (Figure 5(d)). These results suggest that under the effect of HDFs, ASCs may also secrete TGF-*β*1.

Epidermal growth factor (EGF) is also known to stimulate HDF replication and type I collagen accumulation [18]. UVB irradiation reduced the gene and protein expression of EGF. In the UVB-irradiated group, coculture with ASCs promoted HDF protein expression of EGF, but the gene expression did not change significantly. In the nonirradiated group, the protein and gene expression of EGF was significantly increased when HDFs were cocultured with ASCs (Figures 5(a)–5(c)). However, we found that coculture with ASCs induced no significant difference in the expression of EGF in the cell supernatant (Figure 5(d)). We hypothesized that this may be caused by ASC consumption of EGF in the cell supernatant.

UVB-irradiated HDFs had significantly reduced expression of Insulin-like growth factor (IGF)-1 (Figures 5(a)–5(c)). Coculture with ASCs promoted the expression of IGF-1. The gene expression in the UVB-irradiated group was significantly different when the HDFs were cocultured with ASCs, whereas no difference was observed in the nonirradiated group.

### 3.5. Histological Determination of Skin Architecture in Mice

We evaluated the efficacy of ASC treatment to rejuvenate skin in a photoaging mouse model, specifically on reversing wrinkles and increasing thickness and density of the dermis. Histological analyses of skin architecture on week 3 through week 7 postinjection were performed on skin samples harvested from the mice. Histological analysis of the skin showed the effects of ASCs on structural changes and the amount of collagen deposition in the skin. The wrinkles in the UVB-irradiated photoaging group were more superficial and thinner on the ASC-injected side than the PBS-injected side, especially at week 7 (Supplementary Figure 1). The photoaging group had deep, wide wrinkles compared with those of the control group.

We assessed the state of the skin using H&E and Masson's trichrome staining, which showed the quantity of dermal collagen (Figures 2(b) and 2(c)). The epidermal thickness showed no significant differences between the groups over time (Figure 2(d)), but overall, the epidermis was significantly thinner in the UVB-irradiated photoaging group than in the control group (Figure 2(e)). The thickness of epidermal in both groups showed a downward trend in the PBS side, but with no obvious variation trend in the ASCs side. Compared with that of the control group, the thickness of the dermis in the UVB-irradiated photoaging group was significantly reduced, and collagen fibers in the UVB-irradiated photoaging group were abnormal, fragmented, and disorganized. In the ASC-injected side of the UVB-irradiated photoaging group, the thickness of the dermis was significantly increased compared to that of the PBS-injected side at 5 weeks, resulting in the most abundant and dense collagen fibers. In the ASC-injected side of the control group, there was a significant increase in the thickness of the dermis at 6 weeks compared to that of the PBS-injected side (Figures 2(f) and 2(g)). The dermal thickness gradually decreased in PBS-injected side of both groups, while the dermal thickness remained stable in ASC-injected side. There was no significant difference in the thickness of subcutaneous fat between each group (Figures 2(h) and 2(i)), indicating that the injection of ASCs does not contribute to the thickness of the subcutaneous fat layer. There was no regular change trend of the thickness of the subcutaneous fat between the groups.

### 3.6. Expression of MMP-1, TGF-*β*1, EGF, and IGF-1 in Mouse Skin

**Figure 6:**
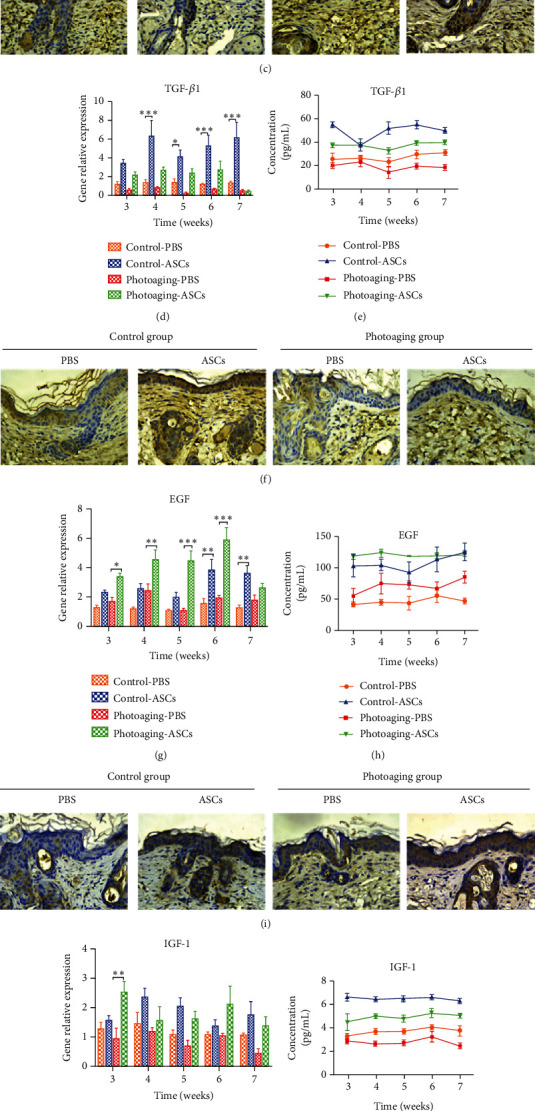
The levels of MMP-1, TGF-*β*1, EGF, and IGF-1 in mouse skin. (a, d, g, j) RT-PCR results showing gene expression in mouse skin. (b, e, h, k) The level of cytokines in skin tissue homogenate. (c, f, i, l) Immunohistochemical labeling of cytokines at 7 weeks.

The expression of MMP-1, TGF-*β*1, EGF, and IGF-1 was detected by PCR, ELISA, and immunohistochemistry in mouse skin. The gene expression of MMP-1 was significantly decreased in the ASC-injected side compared with contralateral controls in the UVB-irradiated photoaging group beginning at 4 weeks. There was no significant difference in MMP-1 gene expression on either side of the control group (Figure 6(a)). ELISA results showed that MMP-1 in skin tissue homogenate was increased in UVB-irradiated photoaging mice skin treated with ASCs, while no difference was found in the control group (Figure 6(b)). As shown in Figure 6(d), the gene expression of TGF-*β*1 drastically increased following ASC treatment in UVB-irradiated photoaging mice beginning at 4 weeks, while there was no difference in either side in the control group. However, the ELISA results showed that TGF-*β*1 expression in the ASC-injected side was significantly higher than that in the PBS-injected side in the both UVB-irradiated photoaging group and control groups (Figure 6(e)).

In the ASC-injected side, the gene expression of EGF was significantly increased at 3 weeks in the UVB-irradiated photoaging group and at 6 weeks in the control group (Figure 6(g)). ASC injection increased the level of EGF in both groups (Figures 6(h) and 6(i)). The gene expression of IGF-1 was not significantly changed over time, but the ELISA and immunohistochemistry results showed that ASC injection significantly increased the level of IGF-1 in the UVB-irradiated photoaging and control groups (Figures 6(j)–6(l)).

### 3.7. Effects of ASCs on Oxidative Stress and Related Factors in Mice

**Figure 7:**
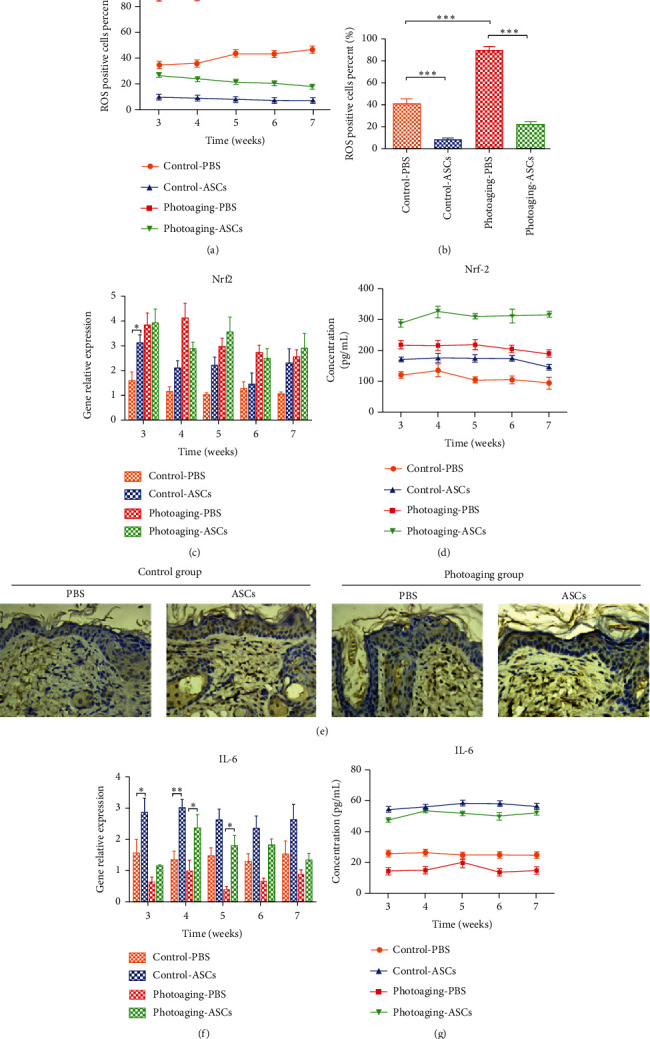
ROS levels and the expression of Nrf-2 and IL-6 in mouse skin. (a, b) The percentage of ROS-positive cells in mouse skin. (c, f) RT-PCR results showing gene expression in mouse skin. (d, g) The level of Nrf-2/IL-6 in skin tissue homogenates. (e) Immunohistochemical labeling of Nrf-2 at 7 weeks.

We determined the percentage of ROS-positive cells in mouse skin. In both groups, ASC injection reduced the level of ROS (Figures 7(a) and 7(b)). There was no difference in the gene expression of Nrf-2 between the ASC-injected and PBS-injected sides in the UVB-irradiated photoaging group and the control group (Figure 7(c)). However, the ELISA results showed that injection of ASCs significantly increased Nrf-2protein concentration (Figure 7(d)). Epithelial keratinocytes also express high levels of Nrf-2, which is critically involved in UV radiation protection [19]. Immunohistochemistry showed that Nrf-2 is mainly expressed in the epidermis (Figure 7(e)), indicating that the increase in the expression of Nrf-2 in the ASC side was mainly caused by increased Nrf-2 in the epidermis. In both the UVB-irradiated photoaging group and control group, IL-6 expression in the ASC-injected side was significantly higher than that in the PBS-injected side (Figures 7(f) and 7(g)). Il-6 is a proinflammatory cytokine, and we hypothesize that the increase in Nrf-2 suppressed IL-6 expression in HDFs [20].

## 4. Discussion

Skin photoaging is mainly caused by UVB irradiation. Skin exposure to UVB radiation can cause various inflammatory cascades in HDFs and different inflammatory cells, leading to skin aging, wrinkling, and hyperpigmentation [21]. Key features of skin aging are thinning of the dermal layer, decreased collagen, and ECM structural changes leading to wrinkles. HDFs are the primary cells in the dermis, but UVB radiation mainly affects the upper layer of HDFs. Some research has shown that ASCs reduce skin wrinkles and reverse aging of the skin [22, 23]; however, the mechanism remains unclear. We found that the paracrine effect of ASCs can better improve the function of HDFs that are not exposed to UVB irradiation than HDFs exposed to UVB radiation, suggesting that ASC regenerative effects may function through lower dermal HDFs.

The quality and quantity of HDFs are significantly reduced in aging skin. Senescent HDFs in the skin have an important effect on age-related skin pathologies and skin cancer [24]. Our research showed that the paracrine effect of ASCs significantly improved HDF senescence, proliferation, apoptosis, and migration in both the UVB-irradiated group and the nonirradiated group. Compared with UVB-irradiated HDFs, nonirradiated HDFs displayed higher proliferative and migration abilities, lower apoptosis, and fewer senescent cells when cocultured with ASCs. These results suggest that under the paracrine effect of ASCs, the number of nonirradiated HDFs was greater, and the cell status was better than those of irradiated HDFs.

One of the most popular aging hypotheses is that skin aging is heavily influenced by external oxidative stress [25]. Oxidative stress is caused by the accumulation of ROS, which can degrade biological molecules, induce cell death and cell apoptosis and stimulate the release of inflammatory factors [26]. Inflammation is considered an important factor in skin aging [27]. Nrf2 is a transcription factor that is activated in response to ROS-induced oxidative stress; thus, Nrf2 is the main defense against oxidative stress-induced damage in the skin [28]. ROS weaken the interaction of Keap1 and Nrf-2, thereby activating Nrf-2 [29]. As expected, we showed that UVB irradiation significantly increased both ROS and Nrf-2 in HDFs. ASCs reduced ROS and activated Nrf-2 in both UVB-irradiated and control HDFs. Some studies have revealed that Nrf2 activation inhibits interleukin-6 (IL-6) induction by opposing the transcriptional upregulation of proinflammatory cytokine genes [20]. IL-6 inhibits human melanocyte proliferation and melanin synthesis to whiten skin [30]. Our results showed that ASCs increased IL-6 expression in UVB-treated and untreated HDFs to above respective baseline levels. As IL-6 has also been shown to inhibit melanocyte proliferation and synthesis of melanin [30, 31], these results may also explain clinical observations of ASC-whitening effects.

HDF reduction in ECM production is another important manifestation of skin aging; thus, we studied some ECM-related cytokines. Cytokines play an important role in the overall cell biology of skin by regulating intracellular signaling pathways. Skin photoaging is caused by the upregulation of MMP1, which is produced by HDFs and induces the degradation of collagen types I, II, and III [32] and the downregulation of TGF-*β*1, which induces collagen synthesis [33]. Collagen is an important extracellular component in human skin that undergoes continuous remodeling and turnover. TGF-*β*1 also inhibits melanin production by melanocytes [34]. ASCs reduce HDF secretion of MMP1 to protect against collagen degradation and increase HDF secretion of TGF-*β*1 to promote collagen synthesis. Interestingly, ASCs promoted the gene expression of TGF-*β*1 in nonirradiated HDFs but not in UVB-irradiated HDFs. TGF-*β*1 could inhibit melanin formation by multiple mechanisms [35, 36]. Therefore, the whitening effect of ASCs may also occur through TGF-*β*1 that is secreted by nonirradiated HDFs. Another kind of adipose-derived cells that showed stem cell characterization is dedifferentiated adipocyte (DFAT), which has the proliferative ability, lineage redifferentiation potential, and pluripotent features [37]. Xu et al. study showed that DFAT is also useful for photoaging skin due to secreting TGF-*β*1 [38]. TGF-*β*1 plays an important role in both ASCs and DFAT antiphotoaging effect. EGF stimulates fibroblast replication with type I collagen accumulation [18]. The gene expression of EGF in UVB-irradiated HDFs increased significantly, but in the nonirradiated HDFs, it showed no significant change. IGF-1 is a molecular marker of aging, and the expression of IGF-1 is reduced with aging [39]. IGF-1 has antiapoptotic effects in many cells [40, 41]. IGF-1 is produced by HDFs in the skin [42], and senescent HDFs secrete insufficient levels of IGF-1, resulting in keratinocytes becoming prone to initiating skin cancer [43]. The reduced expression of IGF-1 in UVB-irradiated HDFs indicating that senescent HDFs were increased and antiapoptotic activity was decreased. Cocultured with ASCs promoted the expression of IGF-1. We demonstrated that paracrine crosstalk between HDFs and ASCs leads to various changes in gene expression and secreted protein levels. These changes in cytokine expression remodel the ECM.

The coculture assay itself represents a simplistic model of the in vivo conditions. Therefore, we designed an animal experiment to further clarify the effect of ASCs. In vivo results showed that the thickness of the dermis differed between the ASC-injected side and PBS-injected side at 5 weeks in the UVB-induced photoaging group and at 6 weeks in the control group. This result indicates that ASCs take at least 5 weeks to exert their effects in animal skin. This result is meaningful for clinical practice in the future. Analysis of the expression of MMP1 and TGF-*β*1 showed that in the UVB-irradiated photoaging group, the reduction in MMP-1 plays a major role in the ASC-injected side, mainly by reducing the degradation of collagen to increase collagen levels and thicken the dermis. In the control group, the increase in TGF-*β*1 plays an important role in the ASC-injected side, mainly by promoting collagen synthesis to thicken the dermis. These results are consistent with our in vitro results showing ASCs induced TGF-*β*1 expression in nonirradiated HDFs. ASCs decreased the expression of ROS to reduce the damage caused by oxidative stress. The antioxidant stress factor Nrf-2 showed no significant difference between groups. Our results show that the level of IL-6 was decreased in UVB-irradiated photoaging mice and control mice. The injection of ASCs increased the level of IL-6. These results are consistent with the in vitro study.

Based on our results, we hypothesize that ASCs repair UVB-induced skin aging by protecting HDFs from apoptosis, reducing ECM degradation, and increasing lower dermal fibroblast proliferation and migration. Under normal conditions, UVB irradiation increases the number of damaged HDFs in the upper dermis. As our in vitro results suggest that ASC paracrine effects are more robust in healthy HDFs than UVB-damaged, ASC-induced skin rejuvenation effects most likely result from changes in this cell population.

**Figure 8:**
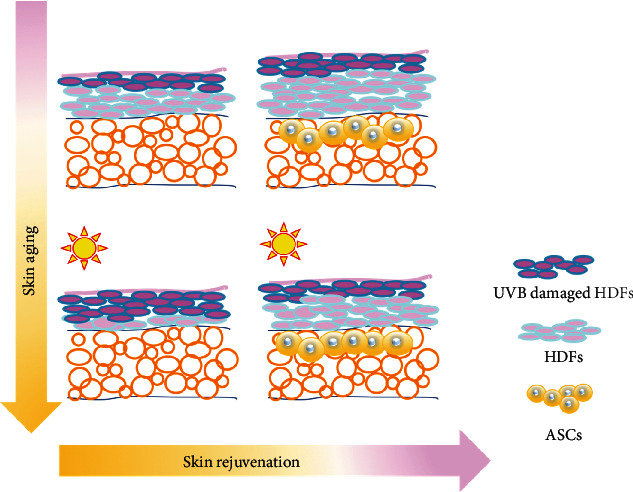
Schematic illustration showing the possible mechanisms by which ASCs improve aging skin through competition between UVB-damaged and undamaged HDFs.

Figure 8 illustrates the possible mechanisms by which ASCs protects against aging in the skin. We consider cell turnover to be crucial for skin youthfulness, homeostasis, quality control, and eventual aging. A limitation of the present study is the lack of evidence showing changes in the proportion of upper-layer UVB-damaged HDFs and lower-layer healthy HDFs in vivo. New markers to distinguish UVB-damaged HDFs and healthy HDFs are needed to clarify this possible mechanism.

## 5. Conclusion

In conclusion, our findings indicate that ASCs can improve the function of both UVB-damaged and normal HDFs through paracrine effects. ASCs coordinate different damaged HDFs by regulating cell proliferation, apoptosis, migration, and cytokine expression. In the ASC-regulated microenvironment, normal HDFs in the lower dermis may be more competitive than UVB-damaged HDFs in the upper dermis. These data provide a new understanding of ASCs in skin rejuvenation. Future research may help clarify whether cell competition plays a key role in ASCs reversing the skin aging process and may lend insight into further clinical applications.

## Data Availability

The analyzed data sets generated during the study are available from the corresponding author on reasonable request.

## Abbreviations

HDFs: Human dermal fibroblasts
ASCs: Adipose-derived stem cells
ECM: Extracellular matrix
UVB: Ultraviolet B
MSCs: Mesenchymal stem cells
ROS: Reactive oxygen species
Nrf2: Nuclear factor erythroid 2-related factor
IL-6: Interleukin-6
MMP1: Matrix metalloproteinase 1
TGF-*β*1: Tumor growth factor-*β*1
EGF: Epidermal growth factor
IGF-1: Insulin-like growth factor-1
SA-*β*-gal: Senescence-associated *β*-galactosidase.

## Supplementary Materials

Supplementary Materials Supplementary Table 1: sequences of primers used in qRT-PCR. (a) Primers used in humans. (b) Primers used in mice. Supplementary Figure 1: photographs of skin from mice in the different groups showing wrinkle formation.
