# Supplementary material for: The Paracrine Effect of Adipose-Derived Stem Cells Orchestrates Competition between Different Damaged Dermal Fibroblasts to Repair UVB-Induced Skin Aging
Source: Stem Cells Int. 2020 Dec 17;2020:8878370. doi: 10.1155/2020/8878370 (PMC7759414; doi:10.1155/2020/8878370)
Supplement: Supplementary Materials — Supplementary Table 1: sequences of primers used in qRT-PCR. (a) Primers used in humans. (b) Primers used in mice. Supplementary Figure 1: photographs of skin from mice in the different groups showing wrinkle formation. [file 8878370.f1.pdf]

A

Gene		Primer sequence	Accession No.
Nrf2	Forward	TCAGCGACGGAAAGAGTATGA	NM_001145412.3
	Reverse	CCACTGGTTTCTGACTGGATGT	
IL-6	Forward	ACTCACCTCTTCAGAACGAATTG	NM_000600.5
	Reverse	CCATCTTTGGAAGGTTCAGGTTG	
MMP-1	Forward	AAAATTACACGCCAGATTTGCC	NM_001145938.2
	Reverse	GGTGTGACATTACTCCAGAGTTG	
TGF-β1	Forward	GGCCAGATCCTGTCCAAGC	NM_000660.7
	Reverse	GTGGGTTTCCACCATTAGCAC	
EGF	Forward	TGGATGTGCTTGATAAGCGG	NM_001178130.3
	Reverse	ACCATGTCCTTTCCAGTGTGT	
IGF-1	Forward	GCTCTTCAGTTCGTGTGTGGA	NM_000618.5
	Reverse	GCCTCCTTAGATCACAGCTCC	
β-actin	Forward	TCCTCCTGAGCGCAAGTACTCC	NM_001101.5
	Reverse	CATACTCCTGCTTGCTGATCCAC	

B

Gene		Primer sequence	Accession No.
Nrf2	Forward	ACTCAAATCCCACCTTAAACAC	NM_010902.4
	Reverse	GTCACAGCCTTCAATAGTCCC	
IL-6	Forward	GACTGATGCTGGTGACAACC	NM_001314054.1
	Reverse	AGACAGGTCTGTTGGGAGTG	
MMP-1	Forward	AGGTTGAGGCTGAGCTCTTT	NM_008607.2
	Reverse	AAGGTCACGGGATGGATGTT	
TGF-β1	Forward	TTGCTTCAGCTCCACAGAGA	NM_009371.3
	Reverse	CAGAAGTTGGCATGGTAGCC	
EGF	Forward	AGAAGGCTACGAAGGAGACG	NM_001310737.1
	Reverse	AGAGTCAGGGCAACTCAGTC	
IGF-1	Forward	CTCTTGGGAGATGCAAAGGC	NM_001111274.1
	Reverse	GTGTGACCCAGGTTGCAAAT	
β-actin	Forward	CTCCTGAGCGCAAGTACTCT	NM_007393.5
	Reverse	TACTCCTGCTTGCTGATCCAC	

Supplementary Table 1. Sequences of primers used in qRT-PCR. *A. Primers used in humans. B. Primers used in mice.*

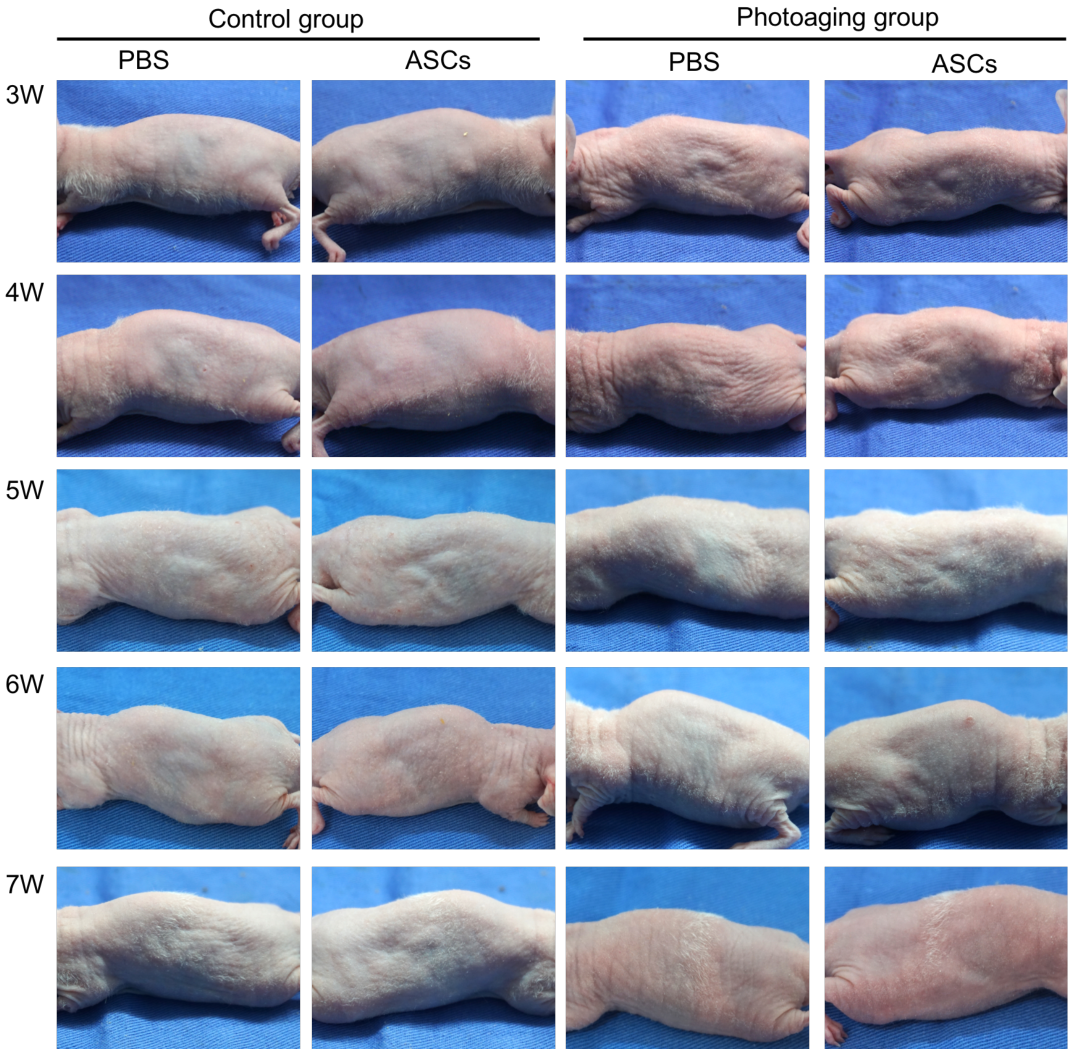

Supplementary Figure 1. Photographs of skin from mice in the different groups showing wrinkle formation.
